# Chemiluminescent Reaction Induced by Mixing of Fluorescent-Dye-Containing Molecular Organogels with Aqueous Oxidant Solutions

**DOI:** 10.3390/gels10080492

**Published:** 2024-07-25

**Authors:** Yutaka Ohsedo, Kiho Miyata

**Affiliations:** 1 Division of Engineering, Faculty of Engineering, Nara Women’s University, Kitauoyahigashi-machi, Nara 630-8506, Japan; 2 Faculty of Human Life and Environment, Nara Women’s University, Kitauoyahigashi-machi, Nara 630-8506, Japan

**Keywords:** chemiluminescence, fluorescent dyes, low-molecular-weight gelator, molecular organogels

## Abstract

Chemiluminescence in solution-based systems has been extensively studied for the chemical analysis of biomolecules. However, investigations into the control of chemiluminescence reactions in gel-based systems, which offer flexibility in reaction conditions (such as the softness of the reaction environment), have only recently begun in polymer materials, with limited exploration in low-molecular-weight gelator (LMWG) systems. In this study, we investigated the chemiluminescence behaviors in the gel states using LMWG systems and evaluated their applicability to fluorescent-dye-containing molecular organogel systems/oxidant-containing aqueous systems. Using diethyl succinate organogels composed of 12-hydroxystearic acid as a molecular organogelator, we examined the fluorescent properties of various fluorescent dyes mixed with oxidant aqueous solutions. As the reaction medium transitioned from the solution to the gel state, the emission color and chemiluminescence duration changed significantly, and distinct characteristics were observed, for each dye. This result indicates that the chemiluminescence behavior differs significantly between the solution and gel states. Additionally, visual inspection and dynamic viscoelastic measurements of the mixed fluorescent dye-containing molecular gels and oxidant-containing aqueous solutions confirmed that the chemiluminescence induced by the mixing occurred within the gel phase. Furthermore, the transition from the solution to the gel state may allow for the modulation of the mixing degree, thereby enabling control over the progression of the chemiluminescence reaction.

## 1. Introduction

Numerous organisms that utilize bioluminescence for various functions exist in nature. Among animals, well-known bioluminescent species include fireflies, marine fireflies, firefly squid, lanternfish, anglerfish, bioluminescent octopuses, and certain bacteria. In the plant kingdom, bioluminescent fungi such as *Mycena lucentipes* and *Omphalotus olearius* are known to exhibit this phenomenon [1,2]. In the bioluminescence of fireflies and marine fireflies, luciferin, a molecule that is highly reactive with oxygen, is oxidized by the enzyme luciferase to form a high-energy dioxetanone intermediate [3]. The decomposition of this dioxetanone intermediate releases strain energy, and this strain energy elevates the electronic state of the resultant oxyluciferin from the ground state to an excited singlet state, which then decays via fluorescence emission [4]. This natural photochemical reaction forms the basis of artificial chemiluminescence.

Chemiluminescence, akin to bioluminescence, involves the generation of an excited-state intermediate through an oxidation reaction, followed by the emission of light as the intermediate returns to its ground state [5]. This phenomenon is extensively employed in the detection of biomolecules, serving as a powerful bioassay technique. Research and development efforts are actively focused on enhancing the performance and sensitivity of chemiluminescent assays and exploring novel methodologies [4,5,6]. However, optimizing chemiluminescence is challenging owing to its complex multistep oxidation reactions and photophysical processes. Consequently, relevant advancements typically revolve around improving existing systems and developing new detection methods.

Meanwhile, new research and development efforts are being undertaken by altering the reaction media of chemiluminescence. Traditionally, chemiluminescence reactions have primarily involved organic solvent/aqueous systems or purely aqueous systems, wherein fluorescent dyes react with oxidant solutions. However, recent studies have reported chemiluminescence performance improvement through the use of gel states as reaction media [7,8]. These studies indicate that the rate at which the fluorescence intensity due to chemiluminescence within polymer hydrogel systems reduces over time is lower than that observed in the case of solution systems. These results suggest that in gel states, such as polymer hydrogels, the high viscosity compared to solutions leads to lower diffusion rates of substances. Consequently, the molecular motion of the dyes is restricted, suppressing fluorescence quenching and thereby prolonging chemiluminescence. This indicates that altering the medium of chemiluminescent reactions from a normal solution to a gel state can modulate the diffusion properties of substances, affecting the reaction behavior. This highlights the potential for reaction control through changes in the reaction medium and presents intriguing new directions for applications such as the analysis of chemiluminescent bioreactions.

Furthermore, mechanical-force-induced chemiluminescence has been detected in palladium complex systems [9]. Chemical reactions induced by mechanical forces have been studied as mechanochemistry [10,11], and chemiluminescence induced by mechanical forces can be understood as mechanoluminescence [12,13]. To investigate the effects of mechanical forces on chemiluminescence further, one can consider increasing the viscosity or gelling the reaction solution in conventional chemiluminescent systems to ensure effective transmission of mechanical forces throughout the reaction system, in addition to exploring new compound systems such as the palladium complex system described above. When gelling chemiluminescent systems, a gel compatible with oxidizer solutions should be selected and incorporated with fluorescent dyes to evaluate chemiluminescent behavior upon mixing. This compatible gel should have physical cross-links rather than covalent cross-links and should mix homogeneously with oxidizer solutions, exhibiting significant deformability under mechanical forces. Such a property is known as thixotropy [14,15,16], which refers to the ability to transition to a sol state under mechanical stress and revert to a gel state after the removal of stress. These characteristics are observed in molecular gels formed from polymeric or low-molecular-weight gelators (LMWGs) [17,18,19,20,21,22,23,24,25,26,27,28] or polymer gelators [29,30,31]. This area has garnered interest not only for elucidating the relationship between the molecular structure of gelators and gel properties but also for developing injectable or spreadable gels for medical and healthcare applications based on thixotropic behavior. In this study, we aimed to explore chemiluminescent systems utilizing mechanical external forces, distinct from previously reported mechanochemical systems. Specifically, we investigated the behavior of chemiluminescence induced by the mixing of molecular gels derived from LMWGs exhibiting thixotropy, which contained fluorescent dyes, with an aqueous solution of an oxidizing agent. By examining this behavior, we assessed the potential of these molecular gels as reaction media for chemiluminescence. This investigation is particularly interesting as it may lead to the control of chemiluminescence by varying the conditions of mechanical force application, such as mixing conditions.

**Figure 1:**
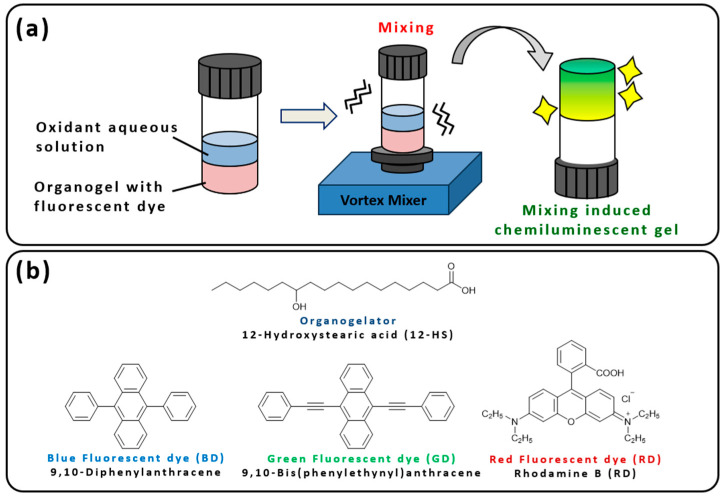
(**a**) Schematic illustration of the research concept of mixing-induced chemiluminescent gel. (**b**) Chemical structures of the organogelator and fluorescent dyes in this study.

Herein, we propose the use of thixotropic molecular gels as reaction media for chemiluminescence to examine the chemiluminescent phenomena induced by mixing molecular gels with oxidant aqueous solutions (Figure 1a). Unlike the conventional mixing of solutions, our proposed approach introduces the mixing of gels and solutions as a novel trigger for chemiluminescence. Specifically, we used 12-hydroxystearic acid (12-HS, Figure 1b) as the LMWG [18,32,33] and ethyl phthalate as the solvent following a simple chemiluminescence system with fluorescent dyes [34]. We decided to use 12-HS because it is an organogelator with a simple molecular structure, which may be less affected by hydrogen peroxide water than other organogelators containing amide or ester bonds, and may effectively maintain the organogel state. First, we investigated the gelation concentrations of organogels required to achieve thixotropy and incorporated fluorescent dyes that emit blue, green, and red light. We then examined the luminescent behavior of these dyes when mixed with oxidant aqueous solutions within the thixotropic molecular organogels, which formed mixing-induced chemiluminescent organogels. Regarding the selection of fluorescent emitting dyes for this study, although this study is a basic investigation of chemiluminescence in gel media, we selected three organic dyes that can cover the visible region and have many examples of research as fluorescent dyes for chemiluminescence, considering their adaptability to various future applications where detection by the human eye is performed. In selecting these organic dyes, we also considered the three primary colors of light (blue, green, and red) as the basis for white-light-emitting gels by mixing the three luminescent colors based on the basic study in this research. The following organic dyes capable of blue, green, and red luminescence were selected from the literature [34] and their chemiluminescence behavior was investigated: 9,10-diphenylanthracene [35], an anthracene derivative of the blue fluorescent dye; 9,10-bis(phenylethynyl)anthracene [36], an anthracene derivative of the green fluorescent dye; 9-(2-carboxyphenyl)-6-(diethylamino)-*N*,*N*-diethyl-3*H*-xanthen-3-iminium chloride (Rhodamine B) [37], a red fluorescent xanthene derivative (Figure 1b).

## 2. Results and Discussion

**Figure 2:**
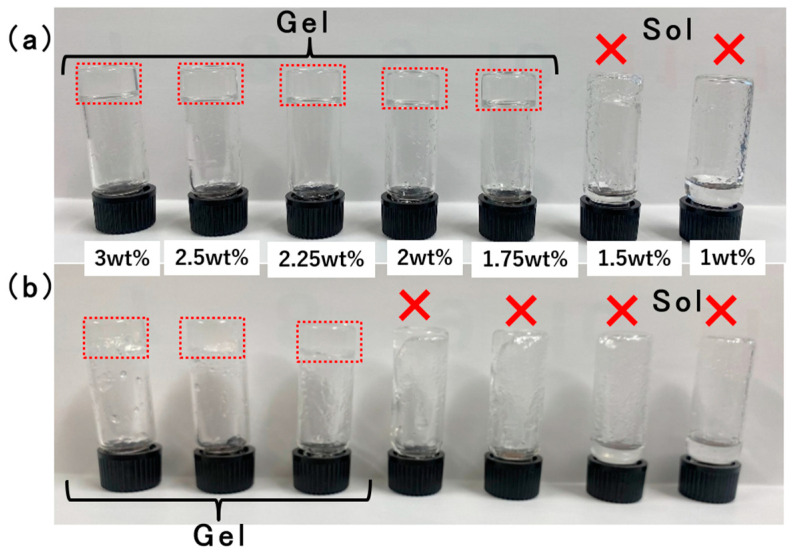
Vial inversion method for the gelation test and thixotropy test: (**a**) Gelation test of 12-HS ethyl phthalate organogels; the solution was heated to dissolve and allowed to cool before inverting the vial. (**b**) Thixotropy test (the vial inversion method for thixotropy) of 12-HS ethyl phthalate organogels; shear force was applied for 10 s in a vortex mixer to bring the vial to a sol state; then the vial was allowed to stand for 1 min before it was inverted.

First, the minimum gelation concentration of 12-HS in ethyl phthalate was determined, and the thixotropic tests of the gel were evaluated using the vial inversion method (Figure 2). The results showed that gelation and thixotropy were confirmed at 2.25 wt% 12-HS. Subsequently, the thixotropic properties of the gels with added dyes were examined. For gels containing blue fluorescent dye (BD, 9,10-diphenylanthracene) and green fluorescent dye (GB, 9,10-bis(phenylethynyl)anthracene), thixotropic behavior was observed at 2.25 wt% 12-HS, whereas for the gel with red fluorescent dye (RD, Rhodamine B), thixotropy was only observed at a higher concentration of 4.5 wt% 12-HS, which was thus selected for further use.

**Figure 3:**
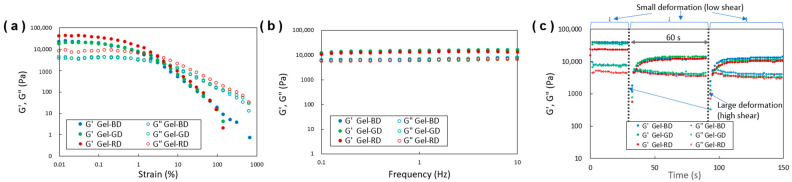
Dynamic rheological properties with respect to (**a**) strain sweep, (**b**) frequency sweep, and (**c**) thixotropic behavior for the ethyl phthalate organogels containing BD, GD, and RD (denoted such as Gel-BD).

The thixotropic properties of the dye-containing gels were quantitatively evaluated through dynamic viscoelastic measurements (Figure 3). Strain sweep tests (Figure 3a) revealed a gel–sol transition, where the storage modulus (G′) exceeded the loss modulus (G″) in the gel state (G′ > G″), and the reverse was observed in the sol state (G′ < G″), confirming the gel state [18,31,38]. Frequency sweep measurements (Figure 3b) also confirmed that G′ remained greater than G″ over a wide frequency range (0.1–10 Hz) [38,39], indicating a stable gel state [40]. To further evaluate the thixotropic properties of the gel’s viscoelastic behavior, intermittent step shear stress was applied (Figure 3c). In Figure 3c, immediately after the application, the samples transitioned to a sol state (G′ < G″); however, it was observed to partially recover to a gel state (G′ > G″) over the course of several tens of seconds. This process was found to be a continuous and reversible transition. Such behavior was exhibited by each gel, and in combination with the results obtained from the inversion method, it was confirmed that the gels in this study exhibit thixotropic properties [14,18].

**Figure 4:**
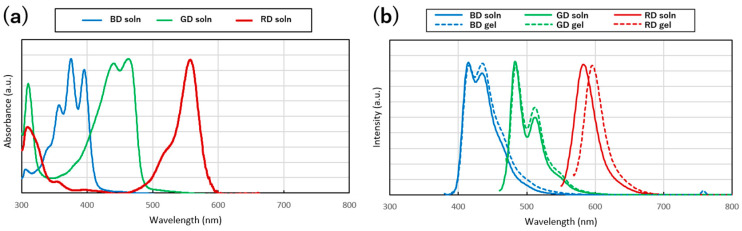
Electronic absorption spectra (**a**) and fluorescent spectra (**b**) of the fluorescent dyes in ethyl phthalate solutions.

The electronic absorption and fluorescence spectra of the dyes in both the gel and solution states were compared (Figure 4). The excitation wavelengths were chosen on the basis of the maximum absorption peaks. The results indicated that compared with the solution state, the emission bands for the gel state exhibited a slight redshift of a few nanometers; however, no significant change was observed in the spectral shapes. This observation suggests that the dyes may be more stably solvated in the gel state than in the solution state [41,42].

**Figure 5:**
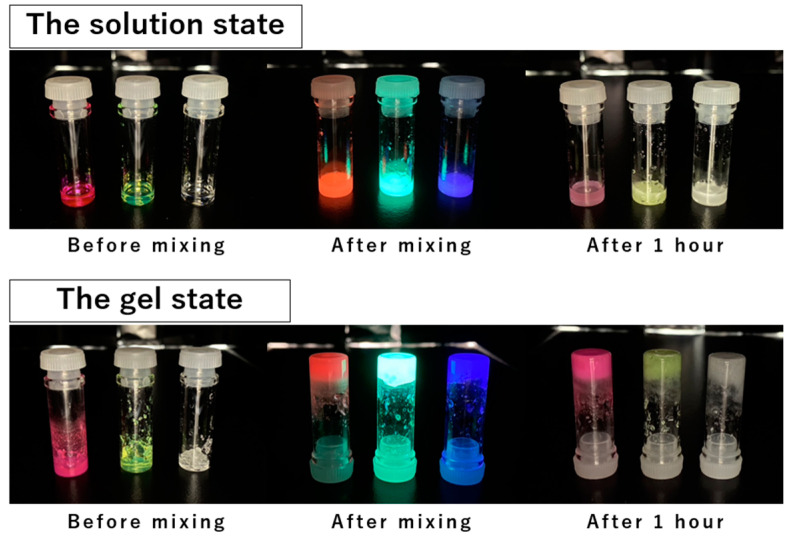
Chemiluminescent spectra after mixing in the solution and gel states.

**Figure 6:**
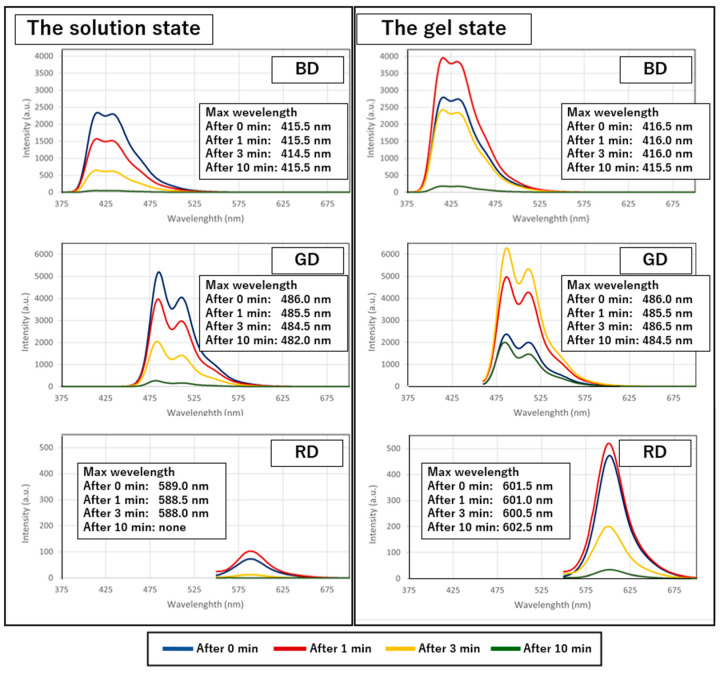
Chemiluminescent spectra after mixing in the solution and gel states.

Next, the chemiluminescence of the obtained fluorescent-dye-containing organogels was investigated by mixing them with an oxidant aqueous solution containing H_2_O_2_. The results showed that upon mixing, the gels and oxidant solution formed homogeneous gel-like mixtures that emitted blue, green, and red light, corresponding to the fluorescent color of the respective dye present in them, as well as the corresponding solution state (Figure 5). Each emission disappeared in approximately 1 h, whereas the mixed gel retained its gel state. The emission spectra, measured using a fluorescence spectrophotometer, were similar to those of the chemiluminescence obtained from mixing fluorescent dye solutions with oxidant solutions, indicating that the observed luminescence originated from the chemiluminescence of the respective fluorescent dyes (Figure 6).

A comparison of the maximum emission wavelengths of the chemiluminescent dyes in the solution and gel states reveals that the maximum emission wavelengths of BD and GD show minimal shifts. However, RD exhibits a significant redshift of approximately 10 nm upon gelation. This observation aligns with the fluorescence spectra measurements, further confirming that RD demonstrates a redshifted emission within the gel medium. The emission can be attributed to the same fluorescent species, although a slight shift is observed over time.

**Figure 7:**
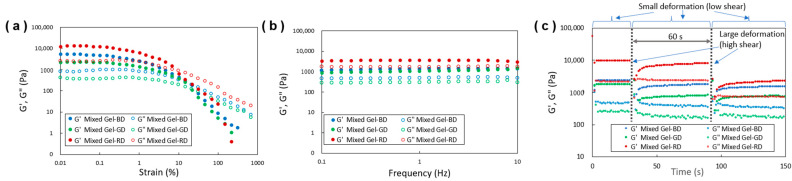
Dynamic rheological properties with respect to (**a**) strain sweep, (**b**) frequency sweep, and (**c**) thixotropic behavior the ethyl phthalate organogels containing BD, GD, and RD with aqueous oxidant solutions (denoted such as Mixed Gel-BD).

The gel state of the mixed gel was confirmed through dynamic viscoelastic measurements (Figure 7), similar to those performed on the gel before mixing. Strain sweep measurements (Figure 7a) indicated a gel–sol transition, which was evidenced by the shift from G′ > G″ (gel state) to G′ < G″ (sol state). Additionally, frequency sweep measurements (Figure 7b) indicated G′ > G″ (gel state) over a wide range in frequency (0.1–10 Hz) as well as also showing in the single gel systems (Figure 3b). These results confirmed that the mixed material maintained a gel state [18,31,38,39,40]. To assess the thixotropic properties of the mixed gel’s viscoelastic behavior, intermittent step shear stress was applied (Figure 7c). Consistent with the behavior of the single-component system described earlier, the samples transitioned to a sol state (G′ < G″) immediately after the stress was applied. Over the course of several tens of seconds, the samples exhibited partial recovery to a gel state (G′ > G″). This transition was observed to be continuous and reversible, though the extent of gel recovery diminished over time. These findings, together with the observations from the inversion method, confirm that the gels in this study demonstrate thixotropic properties [14,18]. It is known that some gelling agents can form macroscopic gel states in W/O systems, where the continuous oil phase holds water droplets and exhibits gel-like mechanical properties [43,44]. Similarly, in O/W systems, some gelling agents allow the continuous water phase to hold oil droplets, also forming a macroscopic gel state with gel-like mechanical properties [45]. These gel materials are expected to be applicable as gel-based excipients in pharmaceuticals. The results of this study, demonstrating the gel state and thixotropic behavior in the diethyl succinate gel/hydrogen peroxide system, indicate that the diethyl succinate gel/water system can maintain its gel structure even in a mixed solvent state. This suggests the possibility that mixed gels of diethyl succinate and water, either in a water-in-oil or oil-in-water type, may exist similarly to the observed results in W/O or O/W gel systems.

**Figure 8:**
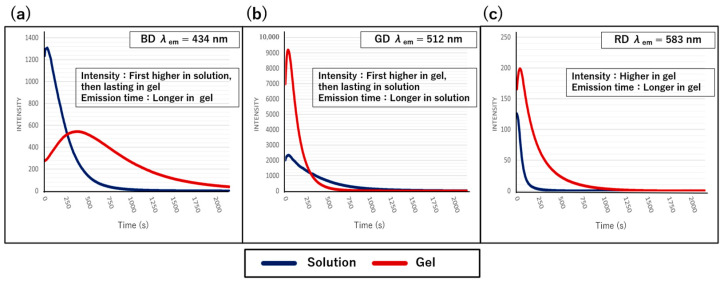
The time-dependent changes in the chemiluminescence intensity. (**a**) BD systems, (**b**) GD systems, and (**c**) RD systems.

Next, time-dependent changes in the chemiluminescence intensity were monitored at the maximum emission wavelength (Figure 8). For BD, the initial emission intensity was higher in the solution state than in the gel state, whereas the emission duration was longer in the gel state than in the solution state. For GD, the initial intensity was higher in the gel state than in the solution state, but the emission duration in the solution state became longer than that in the gel state. For RD, the gel state consistently showed a higher intensity and longer emission duration compared with the solution state. These results indicate that unique differences are observed in the emission intensity and duration based on the type of fluorescent dye and state of the medium (solution or gel) used. Previous studies have reported that in diethyl phthalate solvent, the emission duration is less than one hour, with longer times to quenching observed at lower oxidant concentrations and shorter times at higher concentrations [34]. In our gel system, due to the extended time required for the complete mixing of the gel with the oxidant aqueous solution, the consumption of oxidant takes longer compared to the solution system. Therefore, the gel system may exhibit prolonged fluorescence emission compared to the solution system.

Additionally, in some cases in this study, the emission intensity increases after 1 min (or 3 min) from the initial emission, whereas the chemiluminescence intensity in a solution typically decays over time. This observed increase in the emission intensity suggests that the mixing of the fluorescent-dye-containing organogel and the oxidant aqueous solution is not completed initially and continues to progress over time. While precisely controlling the degree of macroscopic mixing with a vortex mixer is currently challenging, these results indicate the potential for realizing prolonged emission in gel/solution systems. Nonetheless, given the diverse molecular structures of fluorescent dyes, detailed investigations of the relationship between the dye molecular structure and emission behavior in different reaction media are necessary. In this study, we have confirmed the phenomenon of chemiluminescence induced by mixing in the gel state, but the experimental conditions for chemiluminescence have not yet been optimized for the gel system; therefore, future work should focus on exploring and examining organic solvents optimized for the luminescence behavior of individual fluorescent dyes to suppress quenching processes and extend emission duration, aiming to achieve optimal experimental conditions.

**Figure 9:**
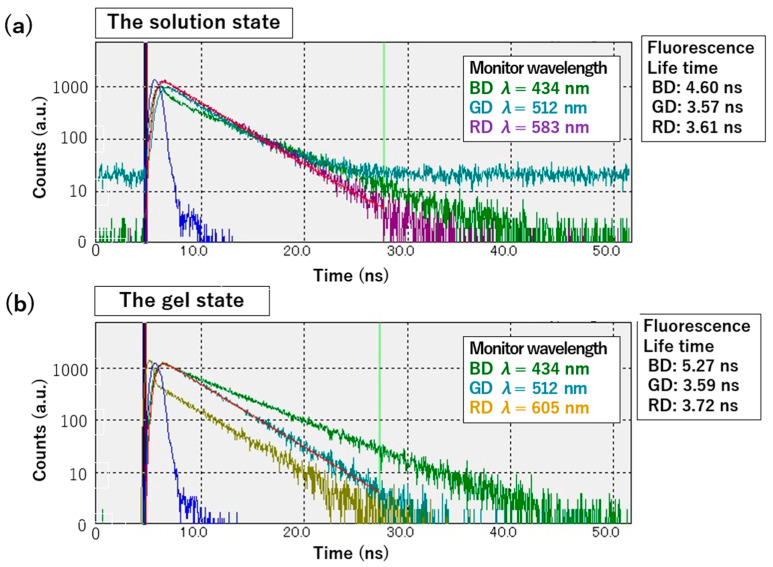
Measured fluorescence lifetime decay in the solution state (**a**) and gel state (**b**). Blue curve: excitation light, red curve: example of fitting curve.

Furthermore, fluorescence lifetime measurements were conducted to evaluate the differences in the dye states between the solution and gel states (Figure 9). For RD and GD, the fluorescence lifetime remained almost unchanged, suggesting that the excited species were the same and that the dye environment remained unchanged. In contrast, the fluorescence lifetime of BD was approximately 1 ns longer in the gel state that in the solution state, indicating a change in the state of the excited species and potentially a different dye environment between the solution and gel states. These fluorescence lifetime results demonstrate that although differences in the fluorescence lifetime due to environmental changes between the solution and gel states are observed for some dyes, no significant differences are detected in the excited state in either condition [46].

## 3. Conclusions

In this study, we investigated the chemiluminescence behavior of LMWG-based molecular gels. Specifically, we examined the chemiluminescent phenomena arising from the mixing of an organogel composed of diethyl phthalate, containing blue, green, and red fluorescent dyes, and LMWG, 12-hydroxystearic acid (12-HS) with an oxidizing aqueous solution. When 12-HS was used as the gelator, the optical properties of the fluorescent dyes changed as the system transitioned from the solution to the gel state. These changes included variations in the emission color and chemiluminescence duration, highlighting distinct characteristics depending on the fluorescent dye. Our findings revealed that the chemiluminescent properties differ between the solution and gel states. Future studies should be focused on investigating various combinations of fluorescent dyes and gel materials to develop novel luminescent organogel materials. For instance, if mixing-induced chemiluminescence from the gel state, as demonstrated in this study, becomes achievable, it could pave the way for applying spreadable thixotropic gels containing fluorescent dyes. By mixing them with an oxidizing agent as needed, chemiluminescence could be generated on-site, potentially offering an alternative to disposable glow sticks known as portable emergency lights that do not rely on external power sources. Furthermore, in this study, we were able to investigate the fluorescence emission behavior induced by chemiluminescence of the primary colors of light—blue, green, and red—in the gel state, and by carefully examining these photochemical and photophysical processes, as well as the energy transition processes, and by controlling the mixing concentration, composition ratio, and mixing process, it is possible to achieve white chemiluminescence in the gel state, opening up the potential for applications in portable emergency lighting.

## 4. Materials and Methods

9,10-Bis(phenylethynyl)anthracene (GD, 98%), 9,10-diphenylanthracene (BD, 98%), bis [3,4,6-trichloro-2-(pentyloxycarbonyl)phenyl] oxalate (CPPO, 98%), and 12-hydroxystearic acid (12-HS, 80%) were purchased from Tokyo Chemical Industry Co., Ltd. (Tokyo, Japan) and used as received. Diethyl phthalate (98%), hydrogen peroxide (33.3 wt% aqueous solution), Rhodamine B (RD, 100%), and sodium acetate (98.5%) were purchased from Wako Pure Chemical Industries, Ltd. (Tokyo, Japan) and used without further purification.

The organogel of diethyl phthalate using 12-HS as a low-molecular-weight organogelator was prepared by placing the appropriate amounts of diethyl phthalate and 12-HS in a vial (4 mL Mighty Vial, Maruemu Corporation, Tokyo, Japan), heating the mixture in a dry bath at 110 °C for 30 min until dissolved, and then cooling it to room temperature for 30 min. The gelation state was evaluated using the inversion method, where the vial was inverted; if the contents did not flow, it was considered a gel, otherwise, it was considered a sol. Thixotropy was also evaluated by applying shear force with a vortex mixer for 10 s to turn the gel into a sol state, allowing the sample to stand for 1 min, and then inverting the vial. If the contents did not flow, it was considered a gel; otherwise, it was a sol (the vial inversion method for thixotropy).

Fluorescent dye-containing molecular organogels were prepared by placing CPPO (800 mg), sodium acetate (100 mg), and 20 mL of 1.0 × 10^−4^ M solutions of BD, GD, or RD in diethyl phthalate along with the required amount of 12-HS (2.25 wt% for BD and GD, 4.5 wt% for RD) to achieve the desired weight percent in a vial (20 mL Mighty Vial, Maruemu Corporation, Tokyo, Japan). The mixture was then heated in a dry bath at 110 °C for 30 min until dissolved and cooled to room temperature for 30 min.

The chemiluminescence behavior of fluorescent dye-containing molecular gels in the presence of an oxidizing aqueous solution was evaluated as follows according to the literature [30]: 111 mg of fluorescent dye-containing organogel (BD, GD, or RD), 2 mg of sodium acetate, and 25 μL of 11 wt% hydrogen peroxide aqueous solution were mixed in a sample tube bottle (Sample-Tube Bottle Clear 2.2 mL, Maruemu Corporation, Tokyo, Japan) and shear force was applied with a vortex mixer for 10 s. The resulting chemiluminescence from the blending of the gel and aqueous solution was observed visually and measured using a fluorescence spectrophotometer (see below). For comparison, the chemiluminescence behavior of the diethyl phthalate solution containing BD, GD, or RD (1.0 × 10^−4^ M) was evaluated under the same conditions.

Dynamic viscoelastic measurements were performed using a Modular Compact Rheometer MCR302e (Anton Paar Japan K.K., Tokyo, Japan) at 25.0 °C, with a gap of 0.5 mm and an 8 mm diameter stainless steel parallel plate. Strain sweep measurements were conducted by fixing the frequency at 1 Hz and varying the shear strain from 0.01% to 100%. Frequency sweep measurements were carried out by fixing the shear strain at 1% and varying the frequency from 0.1 Hz to 100 Hz. Thixotropy (the repeated step-shear measurements) was conducted by a three interval thixotropy test (3ITT) method in Anton paar’s rheometer software [47]. 3ITT is a method to quantitatively evaluate thixotropic properties by measuring a sample’s change in elastic modulus by repeating three intervals: low-shear, high-shear, and low-shear [48,49]. In the present study, the following three intervals were performed: first interval: shear angle gamma = 0.1%, frequency f = 1 Hz for 30 s, second interval: shear rate 3000 s^−1^ for a duration of 0.1 s, third interval: shear angle gamma = 0.1%, frequency f = 1 Hz for a duration of 60 s, and the thixotropic properties of the gels were measured by repeatedly applying the second and third intervals.

UV–Vis absorption spectra were measured using a SEC2020 Spectrometer System 01369 (BAS Inc., Tokyo, Japan) with the solution in a quartz cell with a 1 cm optical path length. Fluorescence spectra were recorded using a Hitachi High-Tech Science F-2500 fluorescence spectrophotometer (Hitachi High-Technologies Corporation, Tokyo, Japan) with excitation wavelengths selected based on the absorption maxima obtained from the absorption spectra of the dye in solution or gel state, and both solution and gel were recorded in a quartz cell with a 1 cm optical path length. Absorption and emission spectra in the solution state were measured using quartz cuvettes (2-face quartz cell for absorption spectra, and 4-face quartz cell for fluorescence spectra). The chemiluminescence fluorescence spectra were measured by placing a mixture of a fluorescent dye-containing gel and hydrogen peroxide solution into a quartz cell under the aforementioned mixing conditions, followed by vortexing the quartz cell prior to measurement.

The fluorescence lifetimes of the fluorescent dye solution and the fluorescent dye-containing gel/hydrogen peroxide mixture (in gel state) were measured using a Quantaurus-Tau Compact Fluorescence Lifetime Spectrometer C16361-01 (Hamamatsu Photonics K.K., Hamamatsu, Japan), with samples placed in a 1 cm path length quartz cuvette. The gel state samples were prepared by mixing the fluorescent dye-containing gel and hydrogen peroxide solution in a quartz cell under the aforementioned mixing conditions and vortexing the quartz cell prior to measurement. For the excitation of the fluorescent dyes, an LED light source was used with excitation wavelengths of 405 nm for BD, 470 nm for GD, and 590 nm for RG. The monitoring wavelength was selected based on the maximum emission wavelength of the fluorescent dyes, and measurements were taken until 1000 counts were reached. The fluorescence lifetimes were calculated by fitting the fluorescence decay curves to a single exponential function.

## Data Availability

The original contributions presented in the study are included in the article, further inquiries can be directed to the corresponding authors.
